# Radiological Patterns of Lung Involvement in Inflammatory Bowel Disease

**DOI:** 10.1155/2018/5697846

**Published:** 2018-08-12

**Authors:** Diletta Cozzi, Chiara Moroni, Gloria Addeo, Ginevra Danti, Monica Marina Lanzetta, Edoardo Cavigli, Massimo Falchini, Fabio Marra, Claudia Lucia Piccolo, Luca Brunese, Vittorio Miele

**Affiliations:** ^1^Department of Radiology, Careggi University Hospital, Florence, Italy; ^2^Department of Experimental and Clinical Biomedical Sciences, Radiodiagnostic Unit No. 2, University of Florence-Careggi University Hospital, Florence, Italy; ^3^Department of Clinical and Experimental Medicine, Internal Medicine and Hepatology, University of Florence, Florence, Italy; ^4^Department of Medicine and Health Sciences, University of Molise, Campobasso, Italy

## Abstract

Inflammatory bowel disease (IBD) is a form of chronic inflammation of the gastrointestinal tract, including two major entities: ulcerative colitis and Crohn's disease. Although intestinal imaging of IBD is well known, imaging of extraintestinal manifestations is not extensively covered. In particular, the spectrum of IBD-associated or related changes in the chest is broad and may mimic other conditions. The common embryonic origin of intestine and lungs from the foregut, autoimmunity, smoking, and bacterial translocation from the colon may all be involved in the pathogenesis of these manifestations in IBD patients. Chest involvement in IBD can present concomitant with or years after the onset of the bowel disease even postcolectomy and can affect more than one thoracic structure. The purpose of the present paper is to present the different radiological spectrum of IBD-related chest manifestations, including lung parenchyma, airways, serosal surfaces, and pulmonary vasculature. The most prevalent and distinctive pattern of respiratory involvement is large airway inflammation, followed by lung alterations. Pulmonary manifestations are mainly detected by pulmonary function tests and high-resolution computed tomography (HRCT). It is desirable that radiologists know the various radiological patterns of possible respiratory involvement in such patients, especially at HRCT. It is essential for radiologists to work in multidisciplinary teams in order to establish the correct diagnosis and treatment, which rests on corticosteroids at variance with any other form of bronchiectasis.

## 1. Introduction

Inflammatory bowel disease (IBD) is a broad term that describes miscellaneous inflammatory diseases of the gastrointestinal tract. The principal pathological entities are Crohn's disease (CD) and ulcerative colitis (UC) [1]. From a pathophysiological point of view, these two conditions are distinct. CD is a relapsing inflammatory disease involving any site of the gastrointestinal tract manifesting with transmural and noncontiguous lesions. UC is an inflammation of the rectum and colon only, characterized by continuous, ascendant alterations that involves only the mucosa, leaving the rest of the colorectal wall unscathed [1–3]. The increasing incidence of IBD in industrialized countries has permitted a better knowledge of these diseases in the last few decades [4]. Extraintestinal manifestations commonly occur in IBD with a prevalence between 21% and 47% [5, 6]. The most common extraintestinal manifestations are cutaneous (*erythema nodosum*, *pyoderma gangrenosum*, and enterocutaneous fistulas), hepatobiliary (primary sclerosing cholangitis, hepatitis, pancreatitis, and portal vein thrombosis), musculoskeletal (arthritis), genitourinary (renal stones, obstructive uropathy), ocular (uveitis, conjunctivitis, and episcleritis), and respiratory (progressive airway and lung involvement, pulmonary vasculature alterations). Radiological imaging plays a fundamental contributory role in detection, characterization, and surveillance of IBD, thanks to computed tomography (CT) and magnetic resonance (MR). Some of the extraintestinal manifestations of IBD can be detected with imaging examinations optimized for the bowel study: these include hepatobiliary, pancreatic, and genitourinary disorders. Further radiological investigations must be warranted if an extraintestinal manifestation is suspected, and it is not possible to study it well with a bowel-targeted examination. In this article, we reviewed the different radiological patterns at high-resolution computed tomography (HRCT) related to respiratory involvement in patients with IBD. Radiologists should be aware of these potential extraintestinal alterations and collaborate with clinicians and reach an accurate diagnosis.

## 2. Respiratory System Involvement in IBD

Respiratory involvement in IBD was first described by Lopez Botet and Rosalem Archer in 1962, and since then, the association has been relentlessly reported [7–10]. The association is rare (prevalence < 1%) and the spectrum of manifestations is broad [5, 6, 9–12]. Current estimates for the prevalence of respiratory abnormality in patients with IBD are around 40%, many of these being subclinical presentations and of uncertain clinical significance, and overall they may be underestimated [8]. Furthermore, in up to 10% patients with IBD, respiratory involvement may be underdiagnosed because their manifestations may precede presentation of bowel disease by months or years [10, 12, 13]. In their review, Black et al. found that IBD patients experience respiratory symptoms with a greater frequency than the general population [8]. Respiratory symptoms may occur more commonly in UC than in CD [10]. Camus et al. noticed that symptoms may develop or worsen after total colectomy in patients with UC [10]. The reason is unknown: the shift of the inflammatory process from the bowel to the lung is often possibly because of the common ancestry of the bowel and the bronchial tree from the primitive gut [10]. Furthermore, additional evidences have to be considered: the parallel flares of bowel and lung symptoms seen in some patients, the steroid sensitivity of the airway disease seen in many patients, and the fact that the biliary tract (which originates from the primitive gut) can also be involved in IBD [10]. Papanikolaou et al. in their study showed a genetic predisposition for various extraintestinal manifestations: haplotypes HLA-A2 and HLA-DR1 in CD and HLA-B27, HLA-B58, and HLA-B8/D3 in UC are more related to joint, skin, and eye disorders [5]. A specific genetic predisposition for respiratory involvement has not been demonstrated yet [5]. The pathogenesis of lung involvement is not yet perfectly known but may relate to a common embryologic origin of respiratory and intestinal mucosa (both derived from the primitive foregut): both the colonic and respiratory epithelia possess goblet cells and submucosal glands. In addition, they both contain submucosal lymphoid tissue and play crucial roles in host mucosal defence. Hypothetically, intestinal and respiratory alterations in these patients could result from epithelial exposure to common antigens by inhalation/ingestion, leading to a sensitization of lymphoid tissue and inflammation. Another physiopathologic theory is that respiratory involvement may be due to inflammatory mediators released by the bowel mucosa [8, 14]. Clinically, patients may present nonspecific respiratory symptoms, such as cough, wheezing, and shortness of breath. Songür et al. found that 16 of 36 IBD patients (44%) in a gastroenterology department had symptoms of wheeze, cough, sputum production, and breathlessness without any evidence of pulmonary infections [15]. Pulmonary function tests (PFTs) are usually suboptimal in patients with IBD in comparison with nonaffected subjects. Furthermore, PFTs may not generally correlate with IBD activity [8, 16]. A number of reports have demonstrated a decrease in diffusion capacity of the lung of carbon monoxide (DLCO) between asymptomatic affected patients and control subjects [15, 17, 18]. Respiratory system involvement includes the airways, lung parenchyma, lung vasculature, and pleural/pericardial serosa (Table 1). The airways may be involved at any level with a wide spectrum of alterations including bronchiectasis, tracheal stenosis, chronic bronchitis, asthma, bronchiolitis, and COPD [8]. Lung involvement patterns in IBD are mainly related to opportunistic infections (which are the main cause, also due to the immunosuppression after IBD-related therapy) and drug-induced toxicity or directly related to primary IBD inflammation [12]. Occasionally, there are concomitant diseases involving the respiratory system in IBD patients, such as asthma, sarcoidosis, chronic obstructive pulmonary disease (COPD), and emphysema related to A1-antitrypsin deficiency (AATD). After arthritis, asthma is the most common comorbidity in both UC and CD, affecting especially males, while sarcoidosis is more common in CD. COPD in IBD patients should be investigated and recognized promptly because it increases the mortality in CD patients, even if it is known that cigarette smoke is a protecting factor against UC (but promotes CD progression) [19]. Furthermore, AATD must be suspected in young patients with IBD and emphysema [5]. Anyway, a clinically oriented approach is necessary, accompanied by targeted radiological diagnostic procedures.

## 3. Role of Imaging

**Figure 1:**
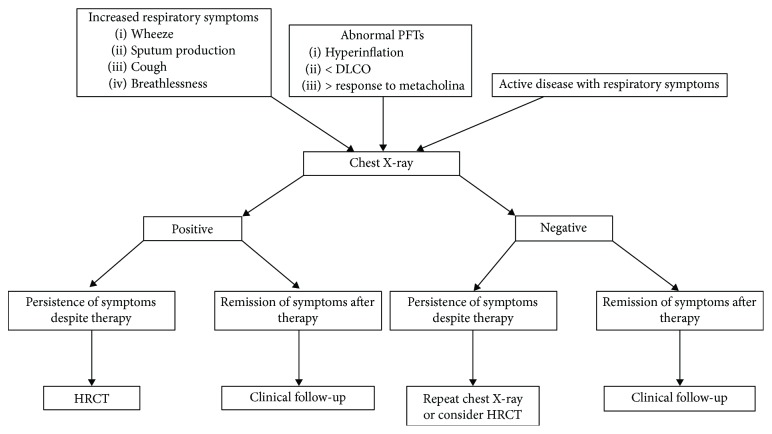
The flowchart indicates when to perform chest X-ray and HRCT in IBD patients.

High-resolution computed tomography (HRCT) has been shown to be the imaging modality of choice for the detection and assessment of adult pulmonary involvement in IBD [6, 8]. HRCT alterations have a prevalence range between 22% and 89%, and radiological findings may be independent of symptoms, pulmonary function tests, and the activity of intestinal inflammation [5, 9]. On the other hand, Songür et al. showed that HRCT abnormalities are present in a large proportion of patients with IBD and approximately 80% of these had active bowel disease [15]. The most common HRCT findings in respiratory involvement include bronchial wall thickening (that may decrease following corticosteroids), bronchiectasis, lung opacities, emphysema, and ground-glass alterations. Recently, HRCT scans at suspended full expiration have been used to study air trapping mainly in patients with asthma, emphysema, bronchiectasis, and constrictive bronchiolitis [15, 20]. Expiratory scans may be also useful for detecting the main causes of mosaic pattern (infiltrative lung disease, small airway disease, and pulmonary vascular disease) [21, 22]. Due to radiation exposure and young age of most IBD patients, HRCT should be used in symptomatic patients in which clinicians have high suspicion of respiratory involvement (Figure 1). Usually, findings at chest X-ray are insensitive and not specific [23]. As chest X-ray is often normal in patients with respiratory symptoms, the radiological patterns remain poorly characterized [10, 23, 24]. The prevalence of pulmonary involvement in children with IBD is low and pulmonary HRCT changes are not common. Therefore, it is not recommended to perform HRCT in children except in those cases with severe clinical respiratory manifestations [25].

## 4. Imaging Features

### 4.1. Airway Disease

**Figure 2:**
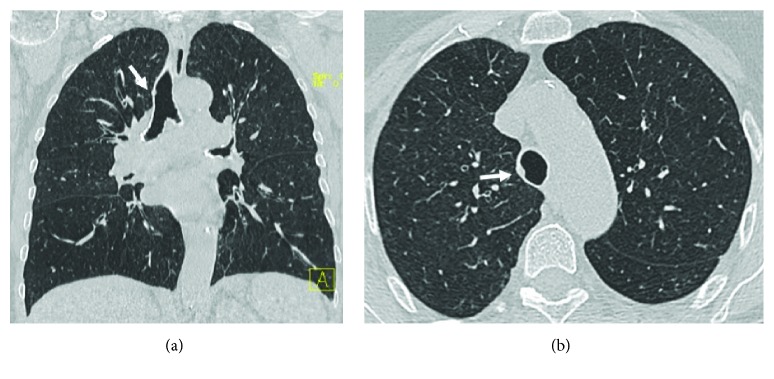
A 77-year-old woman with old history of CD. These HRCT coronal (a) and axial (b) reconstructions show a small nodule in the tracheal wall, most likely referring to a granuloma (arrows).

**Figure 3:**
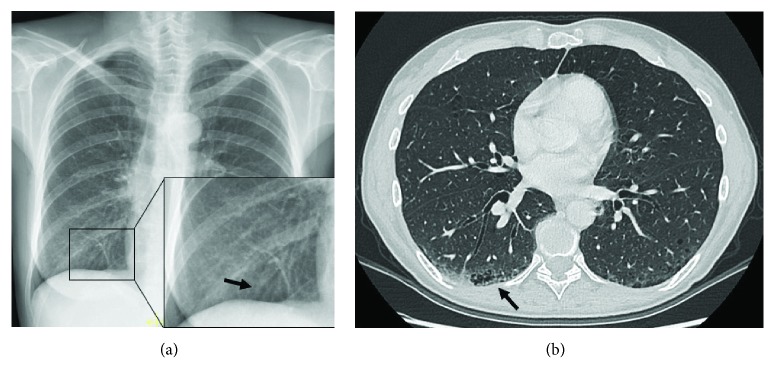
A 55-year-old woman with CD. Chest X-ray shows right basal bronchial wall thickening suggesting bronchitis (a). Axial HRCT study shows peribronchial wall thickening associated with bronchiolectasis (arrow) and signs of subpleural emphysema (b).

**Figure 4:**
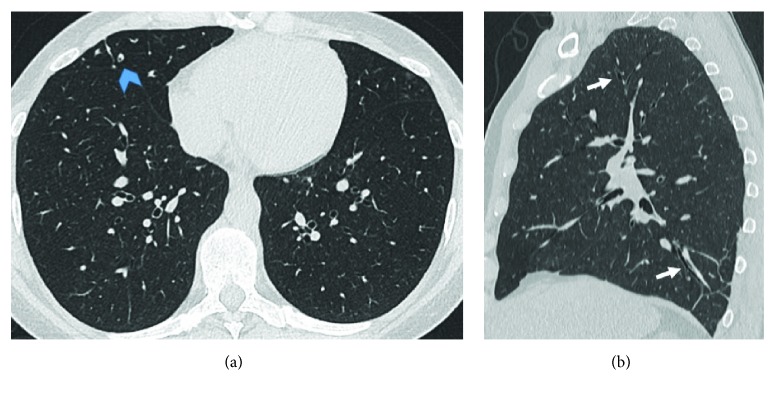
Axial HRCT scan (a) and sagittal reconstruction (b) show bilateral bronchiectasis (arrows) and some small mucoid impactions (blue arrowhead).

**Figure 5:**
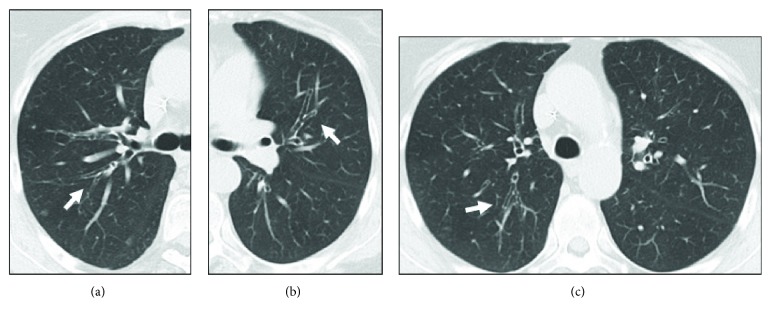
Multiple bilateral bronchiectasis in a 72-year-old woman with CD (arrows). Axial HRCT scans (a, b, c).

**Figure 6:**
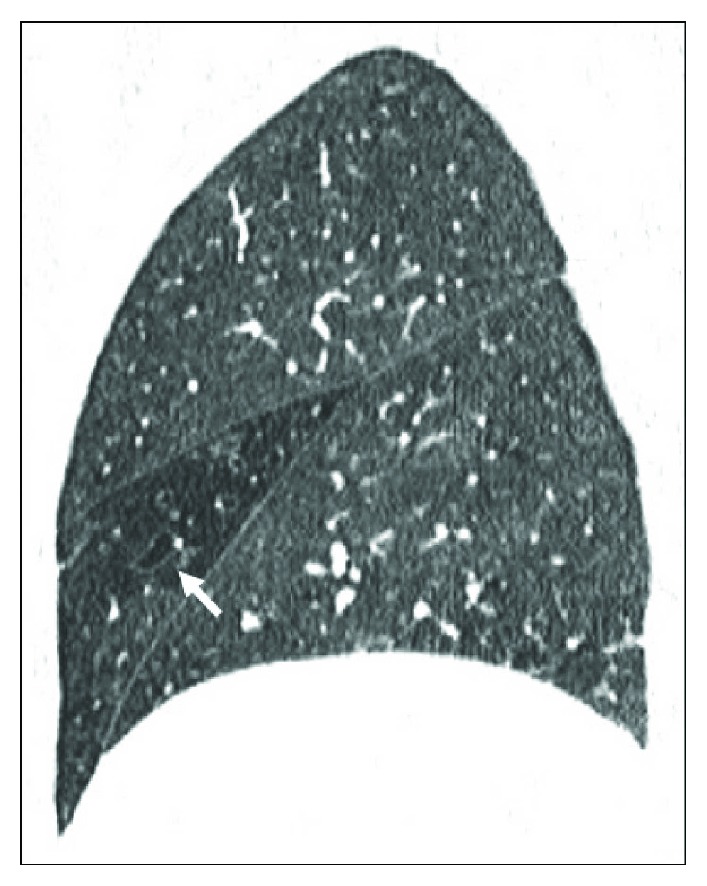
HRCT sagittal reconstruction. Cylindrical and varicose bronchiectasis (arrow) in the middle lobe in a 62-year-old woman with CD. A mosaic pattern due to air trapping is present.

**Figure 7:**
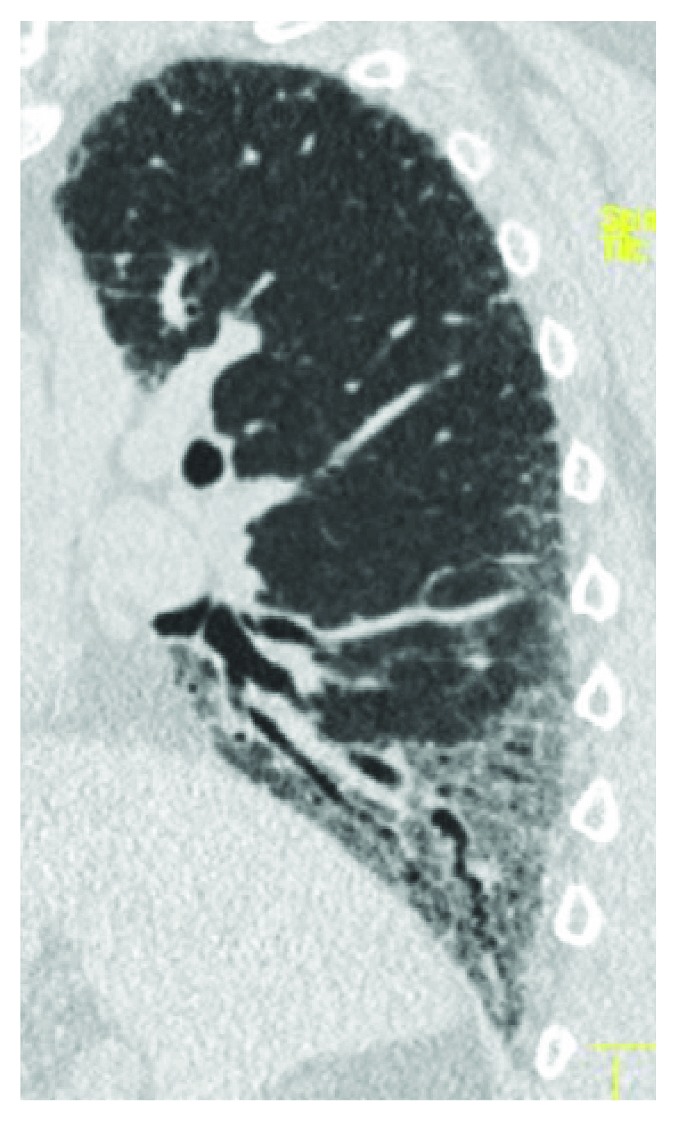
Sagittal HRCT of a 52-year-old male patient with CD shows lower bronchiectasis and bronchiolectasis, associated with diffuse basal parenchymal opacities (ground glass pattern) and interstitial septal thickening.

The most prevalent and distinctive pattern of respiratory involvement in IBD is airway inflammation representing 40–63% of the total of clinically significant respiratory complaints [10]. A wide range of airway abnormalities have been identified in UC patients or less commonly in CD, including subglottic stenosis, chronic bronchitis, and chronic suppurative inflammation of both large and small airways [8, 22]. If left untreated, airway disease can lead to irreversible stenosis in the airway. Upper airway involvement (UAI) in IBD is a rare entity that may involve the pharynx, larynx, trachea, and mainstream bronchi. It may occur up to years after the diagnosis of intestinal bowel disease and in UC can occur after total colectomy [5]. In patients with UC, edema, ulceration, and haemorrhage within the trachea and large airways may mimic the mucosal alterations seen in the colon wall. Instead, tracheobronchitis related to CD has noncaseating and epithelioid granulomas, which may manifest as nodular thickening (Figure 2) [26–28]. Upper airway involvement in IBD may present with cough, hoarseness, stridor, and respiratory distress. Imaging can help in the diagnosis. Chest X-ray is not helpful but sometimes it is possible to show a tracheal narrowing. On the other side, HRCT is able to show definite circumferential or nodular thickening of the tracheobronchial wall. The main differential diagnosis of UAI in IBD includes granulomatosis with polyangiitis (GPA), infections, burns, sarcoidosis, and amyloidosis [29]. Supraglottic, laryngeal, and glottic involvement in patients with sarcoidosis are well recognized, and traction bronchiectasis is often visible in sarcoidosis patients, together with typical mediastinal lymphadenopathy [30]. Respiratory involvement in patients with amyloidosis may occur as part of multisystemic disease or isolated, in case of localized amyloidosis, and the tracheal wall is a frequent site of involvement. Large airways are the most common anatomic site of respiratory involvement in IBD, estimated for about 50% of total respiratory manifestations [5, 26]. Large airway involvement (LAI) is often associated with other extraintestinal manifestations, and it is more common in nonsmoking female with UC. Patients may present with acute/chronic bronchitis, suppurative bronchitis, and bronchiectasis: chronic suppurative airway disease may precede (10%–15%), coexist with (5%–10%), or follow the development of inflammatory bowel disease (8%–85%) [5, 8, 10]. Of particular interest is the fact that, unlike other causes of bronchiectasis, chronic suppurative airway disease associated with UC frequently responds to treatment with inhaled steroids. Chest X-ray may show increased interstitial markings, a reticular pattern, or may appear normal (Figure 3). HRCT classical pattern is characterized by diffuse or sporadic concentric bronchial wall thickening (with or without mucoid impaction) and bronchiectasis (cylindric, varicose, or cystic) (Figures 4 and 5) [12, 31]. Mahadeva et al. demonstrated that 13 out of 17 patients (76%) had bronchiectasis on HRCT [23]. Small airway involvement (SAI) is rarely encountered clinically (3%–10%). It occurs at younger age and tends to present at an earlier point in the disease course than LAI [32–34]. However, with more widespread HRCT use, the detection of SAI in IBD patients is increasing [8]. Chest X-ray can be normal or shows increased lung volumes consistent with air trapping and/or reticulonodular or ground-glass opacities. The typical pattern on HRCT is bronchitis/bronchiolitis characterized by bronchiolar wall thickening, mucoid impaction, centrilobular ground-glass nodules, tree in bud, and mosaic due to air trapping (Figure 6). Chronic bronchiolitis may lead to the progressive bronchiectasis and bronchiolectasis (Figure 7). Inhaled/systemic steroids are the therapy of choice in large and small airway disease in patients with IBD; rarely, immunomodulators, such as TNF-alpha, have been used, because of their limited clinical evidence for efficacy [32, 35–41]. Details of therapy are available in the article by Camus and Colby [42]. At times, bronchial thickness may decrease while steroids are given [43].

### 4.2. Lung Parenchymal Involvement

Lung parenchymal involvement in IBD can be due to nonspecific infection, while true IBD-related interstitial lung disease is rare [8, 10, 12]. Not infrequently in patients who are not postcolectomy, the IBD drug toxicity is involved. Many of the HRCT patterns found in these patients are not pathognomonic for IBD and can be present in more than one context: infection, drug toxicity, or IBD-related changes [44].

**Figure 8:**
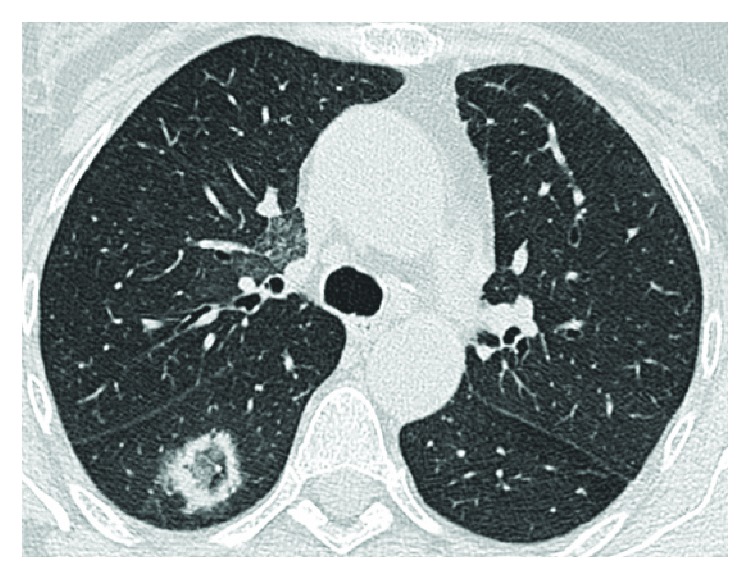
Reverse halo or atoll sign in a patient with UC and possible OP.

**Figure 9:**
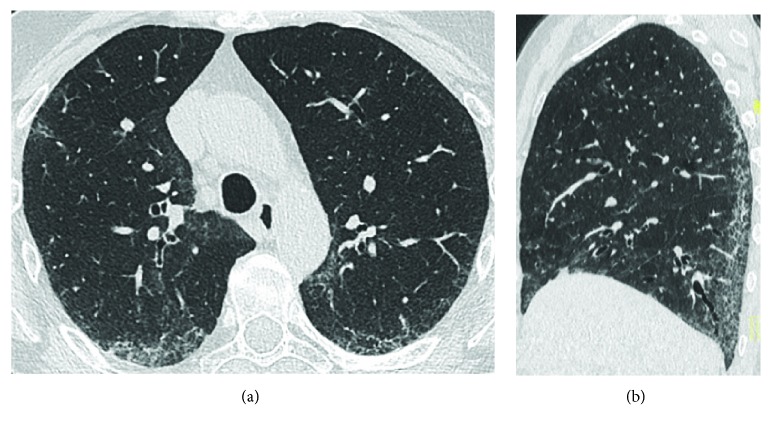
A 67-year-old woman with CD. In both basal posterior segments of the lower lobes, an interlobular-intralobular interstitial thickening associated with ground glass opacities and some traction bronchiectasis and bronchiolectasis is appreciable. A subpleural sparing is associated. These findings are compatible with NSIP. Axial HRCT study (a) and sagittal reconstruction (b).

**Figure 10:**
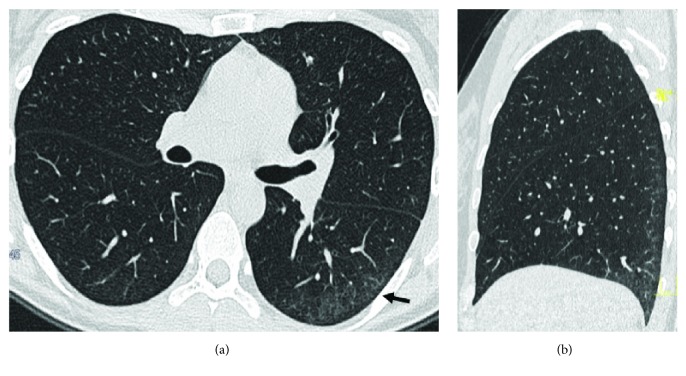
A 36-year-old woman with UC and previous total colectomy. In the basal posterolateral segment of lower lobes (more evident in the left), a fine interstitial thickening associated with ground glass opacities and some traction bronchiolectasis is appreciable. A subpleural sparing is associated (arrow). These findings are consistent with NSIP. Axial HRCT study (a) and sagittal reconstruction (b).

It may be difficult for the radiologist alone to differentiate the exact aetiology, and a multidisciplinary approach to lung alterations in IBD is recommended. Ulcerative colitis is more often reported to be related with parenchymal involvement as opposed to Crohn's. Age of onset is variable and there is a female predominance. Parenchymal disease usually manifests with consolidations consistent with an organizing pneumonia (OP) patterns: although an OP pattern may also develop with infection or occur as a manifestation of drug toxicity, some authors attributed cases related to IBD itself [8, 10, 45]. Chest X-ray shows focal or diffuse peripheral predominant opacities and air bronchograms. HRCT shows patchy, asymmetric, unilateral, or bilateral foci of consolidation in a peripheral or peribronchovascular distribution, large irregular nodules, or ill-defined centrilobular nodules; ground-glass opacities and crazy paving are other characteristics. A “reverse-halo sign” (or “atoll sign”) is considered to be suggestive, although it is seen only in 20% of patients with OP (mainly seen during the OP healing/recovery phase) (Figure 8) [46]. Another parenchymal disease in IBD is cellular nonspecific interstitial pneumonia (cellular NSIP) that has different prognostic values compared to fibrotic NSIP (the former responds to treatment and has a better prognosis). Typical cellular NSIP findings at HRCT are ground-glass opacity with tiny reticulation and a subpleural and basal distribution. A sparing of the immediate subpleural lung is particularly suggestive of NSIP (Figure 9). Honeycombing is typically absent. Except for the colectomized UC patient who is tacking no medication, any discussion regarding interstitial lung disease in IBD should include the patient's drug history. In fact, NSIP is a common pattern of lung drug toxicity (Figure 10) [46]. In particular, sulfasalazine and mesalazine are used in the management of IBD and both can produce pulmonary toxicity that is usually expressed as NSIP, OP, or eosinophilic pneumonia [10, 47, 48]. Patients with a eosinophilic reaction show a combination of lung abnormalities on imaging and increased serum eosinophils. HRCT may show a peripheral, upper lobe predominant consolidation associated with patchy bilateral-nodular or oval mass-like consolidation. These characteristics may closely resemble or mimic OP, but eosinophilic pneumonia often shows an upper lobe predominance instead of OP, where lung bases are most involved.

**Figure 11:**
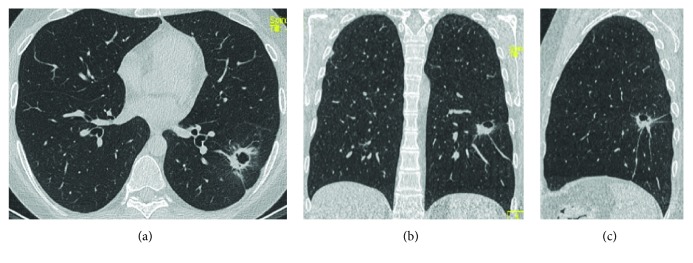
A 55-year-old woman with coexisting CD and GPA disease. These HRCT images (axial (a), coronal (b), sagittal (c)) show a pulmonary necrotic nodule in the lower left lobe: it is possible to notice the necrotic cavity inside. Whether this is due to CD of GPA was unclear.

Pulmonary necrobiotic nodules have also been seen in patients with IBD and should be differentiated from malignancies and an infection. Histologically, these lesions have been reported to be mainly necrobiotic (25%) or granulomatous (12.5%) [8]. On imaging, these nodules are round and well-defined, sometimes cavitated, and can measure up to a few centimetres in diameter (Figure 11). An infection should be excluded because these nodules respond to steroids but not to antibiotics [10, 12]. Any patient on anti-TNF therapy may develop opportunistic infections or tuberculosis [47]. As for the airways, it is also important to remember that occasionally there are concomitant diseases involving lung parenchyma, such as sarcoidosis and emphysema related to AATD [30]. Further investigations are still needed to better elucidate these connections.

### 4.3. Vascular Manifestation

**Figure 12:**
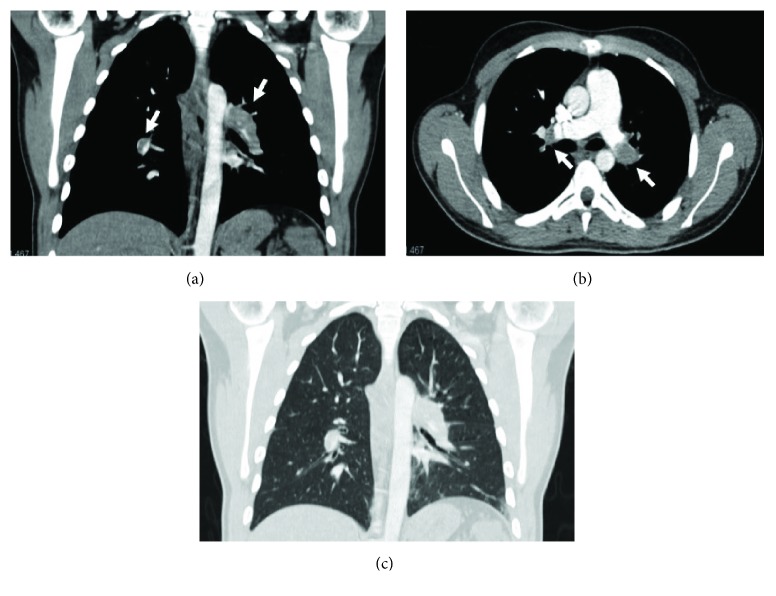
An 18-year-old male with Crohn's disease in the acute phase and no additional comorbidities, who presented with mild shortness of breath. Coronal and axial reconstruction of contrast-enhanced chest CT reveals bilateral pulmonary embolism in both main pulmonary arteries (arrows, a-b). Coronal reconstruction for lung parenchyma (c).

Vascular disease rarely occurs in IBD. They may be represented by granulomatosis with polyangiitis (GPA), microscopic polyangiitis, eosinophilic granulomatosis with polyangiitis (EGPA), and other pulmonary vasculitis non-ANCA+ that have been reported by some authors [49–52]. As venous thromboembolism occurs frequently in IBD patients, acute pulmonary embolism remains the most clinically important pulmonary vascular manifestation in these patients (Figure 12) [6, 8]. In fact, it is known that these patients have increased risk of thromboembolic disease: Bernstein et al. argued that the risk is three times higher compared with age-matched control subjects [53]. Thromboembolic risk in IBD patients may be due to the pathology itself and its related conditions such as abdominal surgery, immobility, drugs, and frequent central venous catheterization [8]. Risk may be further increased during the activity phases of IBD, but up to one-third of thrombotic events occur while the disease is quiescent, suggesting ongoing thrombotic risk unrelated to disease activity or to eventual therapy [54].

### 4.4. Serosal Involvement

**Figure 13:**
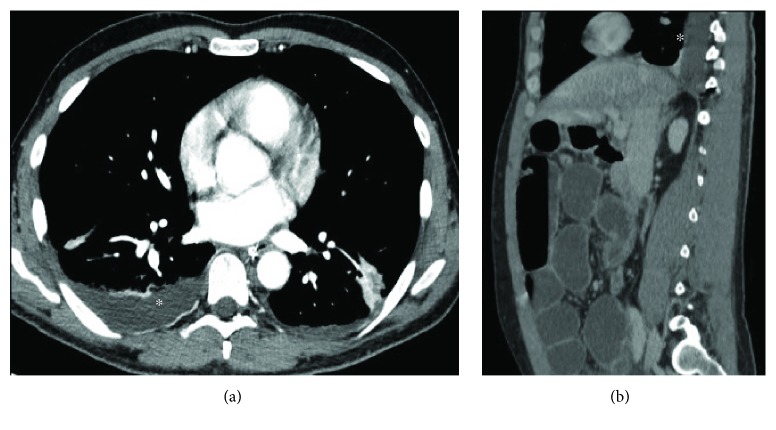
A 43-year-old man with Crohn's disease subjected to previous surgical ileal resection. Asterisks (^∗^) show unilateral pleural effusion during acute abdomen due to intestinal obstruction. Axial CT study (a) and sagittal reconstruction (b).

Pleural and pericardial involvement is rare in IBD [6, 8, 10, 12]. Pleural effusion is nearly always unilateral and exudative in nature (Figure 13). When the pericardium is involved, it is usually not accompanied by pleural effusion: concomitant pleural and pericardial involvement is rare [8, 10, 12, 55]. Serositis may be associated with intense chest pain and the association with myocarditis is possible [10].

## 5. Conclusions

Nowadays, increasing evidence suggests that IBD and extraintestinal manifestations are not isolated diseases but may have common pathophysiological pathways. With the increased use of HRCT, pulmonary manifestations of IBD are more commonly encountered. Bronchiectasis is the most common alteration seen among patients with IBD but the spectrum of respiratory involvement is wide, from organ to specific manifestation. Pulmonary involvement in inflammatory bowel disease should be considered in all patients, even when gastrointestinal symptoms are minimal or well-controlled. Radiologists must be aware that they have the key role to optimize clinical management of IBD patients, escaping from errors and vain therapies, in order to avoid destructive airway changes or irreversible pulmonary fibrosis.

## Figures and Tables

**Table 1:** Respiratory and heart involvement in IBD: sites and patterns. −: not described; ±: unusual; +: occasionally observed; ++: relatively common.

Site of involvement	Pattern	UC	CD
Upper airway	Glottic and subglottic edema/stenosis	++	+
	Isolated tracheal inflammation/bulging	++	+

Large airways	Bronchiectasis	++	+
	Suppurative airway disease	++	±
	Acute bronchitis	+	±
	Chronic bronchitis	++	±

Small airways	Diffuse panbronchiolitis	+	−
	Granulomatous bronchiolitis	−	+
	*Bronchiolitis obliterans* syndrome	+	−
	Necrotizing bronchiolitis	+	++

Lung parenchyma	OP	++	±
	Eosinophilic pneumonia	+	±
	NSIP cellular	+	++
	DIP	+	−
	Fibrosing alveolitis	±	±
	Sterile necrobiotic nodules	+	+

Pulmonary vasculature	Pulmonary embolism	±	±
	Granulomatosis with polyangiitis (GPA)	+	+
	Eosinophilic GPA (EGPA)	±	+
	“Pulmonary vasculitis”	+	+

Serosal surface	Pleural effusion	+	+
	Pericardial effusion	+	+

Concomitant diseases involving lung/airways	Sarcoidosis-like	+	+
	A1-antitrypsin deficiency	+	+
	COPD	+	+
	Amyloidosis	±	±

Various patterns	Pneumomediastinum	±	±
	Pulmonary interstitial emphysema	+	+

Heart/pericardial involvement	Myocarditis	+	+
	Atrioventricular block	+	+
	Pericardial tamponade	+	+
